# Impaired Virus Clearance, Compromised Immune Response and Increased Mortality in Type 2 Diabetic Mice Infected with West Nile Virus

**DOI:** 10.1371/journal.pone.0044682

**Published:** 2012-08-31

**Authors:** Mukesh Kumar, Kelsey Roe, Pratibha V. Nerurkar, Madhuri Namekar, Beverly Orillo, Saguna Verma, Vivek R. Nerurkar

**Affiliations:** 1 Department of Tropical Medicine, Medical Microbiology and Pharmacology, John A. Burns School of Medicine, University of Hawaii at Manoa, Honolulu, Hawaii, United States of America; 2 Pacific Center for Emerging Infectious Diseases Research, John A. Burns School of Medicine, University of Hawaii at Manoa, Honolulu, Hawaii, United States of America; 3 Laboratory of Metabolic Disorders and Alternative Medicine, Department of Molecular Biosciences and Bioengineering, College of Tropical Agriculture and Human Resources, University of Hawaii at Manoa, Honolulu, Hawaii, United States of America; Washington University, United States of America

## Abstract

Clinicoepidemiological data suggest that type 2 diabetes is associated with increased risk of West Nile virus encephalitis (WNVE). However, no experimental studies have elucidated the role of diabetes in WNV neuropathogenesis. Herein, we employed the *db/db* mouse model to understand WNV immunopathogenesis in diabetics. Nine-week old C57BL/6 WT and *db/db* mice were inoculated with WNV and mortality, virus burden in the periphery and brain, and antiviral defense responses were analyzed. *db/db* mice were highly susceptible to WNV disease, exhibited increased tissue tropism and mortality than the wild-type mice, and were unable to clear the infection. Increased and sustained WNV replication was observed in the serum, peripheral tissues and brain of *db/db* mice, and heightened virus replication in the periphery was correlated with enhanced neuroinvasion and replication of WNV in the brain. WNV infection in *db/db* mice was associated with enhanced inflammatory response and compromised antiviral immune response characterized by delayed induction of IFN-α, and significantly reduced concentrations of WNV-specific IgM and IgG antibodies. The compromised immune response in *db/db* mice correlated with increased viremia. These data suggest that delayed immune response coupled with failure to clear the virus leads to increased mortality in *db/db* mice. In conclusion, this study provides unique mechanistic insight into the immunopathogenesis of WNVE observed in diabetics and can be used to develop therapeutics for the management of WNVE among diabetic patients.

## Introduction

It is known that people with type 2 diabetes have higher incidence of bacterial and fungal infections [1]–[3]. Recent studies among diabetic humans and animal models have demonstrated that the dysfunctional innate and adaptive immune responses in the diabetics contribute to increased susceptibility to pathogens such as *Staphylococcus aureus*, *Listeria monocytogenes, Porphyromonas gingivalis,* and *Trypanosoma cruzi* [4]–[6]. These immunological changes include altered levels of specific cytokines and chemokines, and changes in the number and activation state of various leukocyte populations [3], [7]–[9]. Diabetes also inhibits important aspects of leukocyte function, such as chemotaxis and phagocytosis, oxidative burst, and intracellular killing [1], [3]. Defects in adaptive immunity in diabetics include reduced lymphocyte proliferation and delayed type hypersensitivity reactions (Th1-type responses) [3], [7].

West Nile virus (WNV), a neurotropic flavivirus, has emerged as a significant cause of viral encephalitis in the United States. WNV infection in humans is usually asymptomatic or self-limiting, with a mild febrile illness, but may progress to meningitis, encephalitis, paralysis, and death [10]. WNV-associated encephalitis (WNVE) occurs more frequently in persons with compromised immune system, older age and having underlying conditions such as hypertension and type 2 diabetes [10], [11]. Presence of diabetes is a significant risk factor for developing severe WNV disease or death rather than West Nile fever (WNF) [11]–[13]. Patients with diabetes are four times more likely to develop WNVE than WNF, which is significantly more than other underlying conditions such as old age, male gender and hypertension [14], [15]. Moreover, Abroug et al have reported that a higher proportion of patients with WNV infection had hyperglycemia on admission to the hospital [16], and persons with diabetes are most likely to have persistent symptoms after WNV infection [17]. Further, diabetes is also associated with the development and severity of chorioretinitis in patients with WNV infection [18], [19]. These clinicoepidemiological data suggest that type 2 diabetes is associated with increased risk of WNVE. However, no experimental studies have been conducted to decipher the role diabetes plays in WNV disease severity.

WNVE is characterized by disruption of the blood–brain barrier (BBB), neuroinflammation, microglial activation and loss of neurons [20], [21]. An intact innate and adaptive immune response is required to limit WNV infection. Antiviral type I interferon (IFN-α and β) production is essential in suppressing viral titers in the brain and peripheral organs [22]. The induction of WNV-specific immunoglobulins (IgM and IgG) is essential for suppressing viremia and virus dissemination [23], [24]. T-cell-mediated immunity is essential for controlling WNV infection in the central nervous system (CNS). Absence of functional CD8^+^ or CD4^+^ T cells results in failure to clear WNV from infected neurons in the CNS [25], [26]. WNV also induces a dramatic increase in several pro-inflammatory cytokines such as tumor necrosis factor alpha (TNF-α) and interleukin (IL)-1β and -6 [20], [21], [27] and chemokines such as MCP-1 and IP-10, which regulate leukocyte trafficking into the brain, and neuronal death after infection [27]–[29].

In the present study we inoculated the wild-type (WT) and diabetic (*db/db)* mice with WNV and evaluated the course of the ensuing infection, as well as the resultant host antiviral immune response to infection in a quest to develop a diabetic mouse model to test therapeutic options for management of WNVE in diabetics.

## Results

### *db/db* mice have significantly high body weight and are glucose intolerant

**Figure 1:**
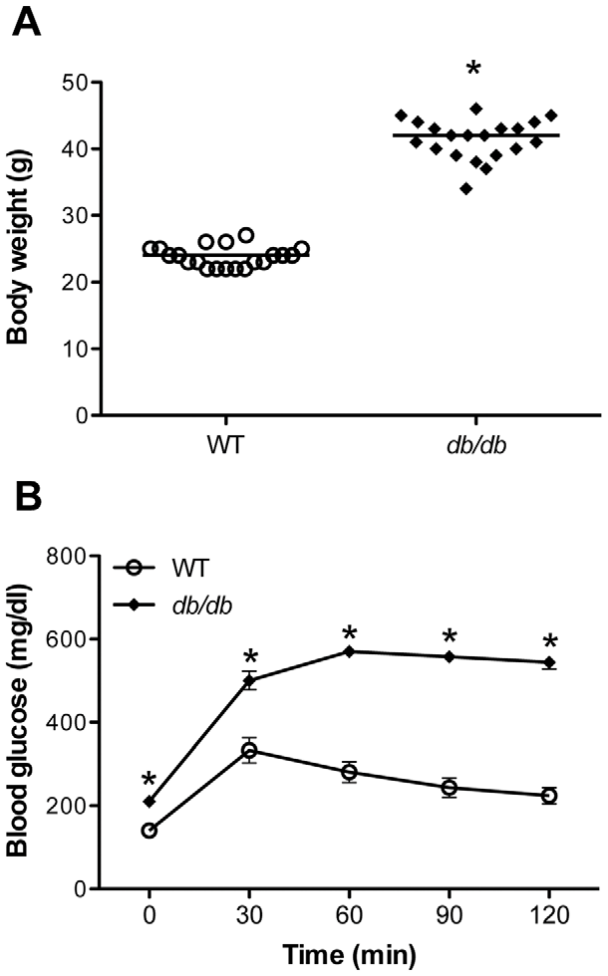
Body weight and intraperitoneal glucose tolerance test in WT and *db/db* mice. (A) Body weight in grams of nine-week old WT and *db/db* mice. (B) Blood glucose levels in the WT and *db/db* mice during IGTT. The data expressed are the mean concentration (mg/dL) ± SEM of the blood glucose levels and are representative of two independent experiments. *p<0.0001.

*db/db* mice, a well defined type 2 diabetes mouse model with mutations in the leptin receptor gene was used to characterize the relationship between diabetes and WNV disease severity [30]. *db/db* mice are the most commonly used model to study the effect of diabetes on various viral, bacterial and parasitic diseases [4]–[6]. As expected *db/db* mice had significantly high body weight as compared to WT controls, 41.4±5.3 vs. 24.3±2.4 g, p<0.0001 (Fig. 1A). *db/db* mice were glucose intolerant as measured by intraperitoneal glucose tolerance test (IGTT) and the blood glucose levels were significantly higher (p<0.0001 for all time points) than those of WT mice (Fig. 1B).

### Susceptibility of *db/db* mice to WNV disease

**Figure 2:**
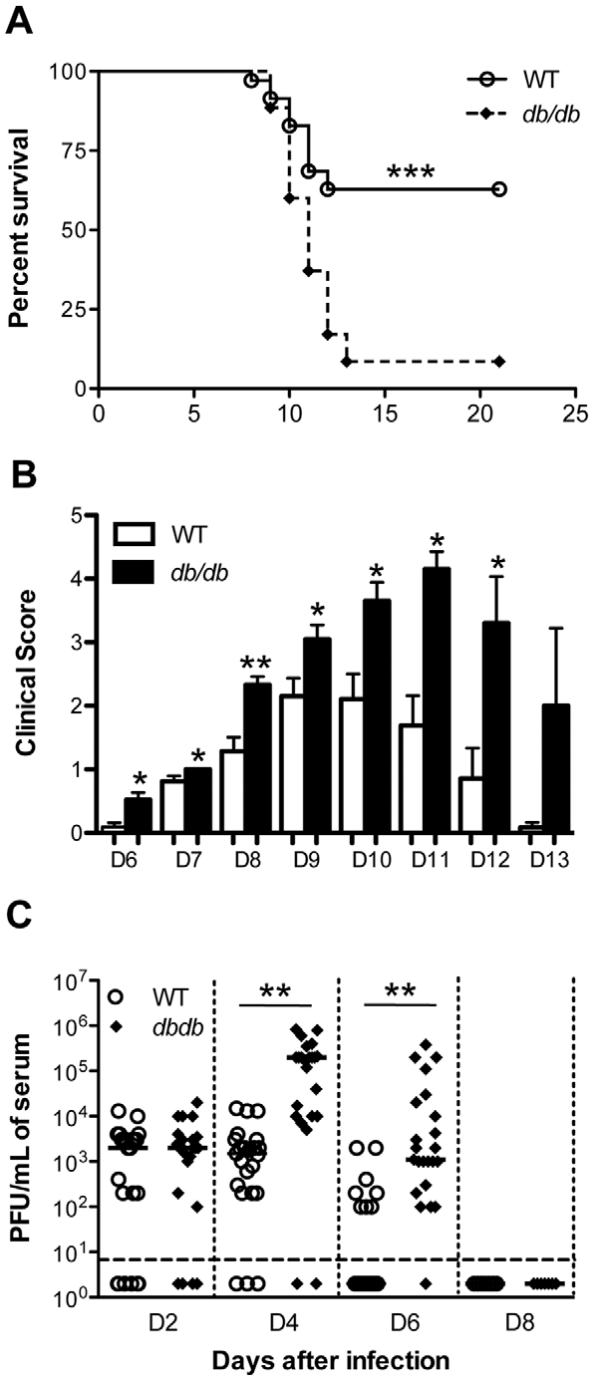
Survival and virological analysis in WT and *db/db* mice after WNV infection. (A) Nine-week old male WT and *db/db* mice were inoculated subcutaneously with 10 PFU of WNV. All mice were observed for 21 days. Data are combined of two independent studies (n = 38 per group). The survival difference between WT and *db/db* mice was statistically significant. All surviving animals were positive for anti-WNV IgG antibodies. (B) Animals were monitored for clinical scores twice a day. The designation for the clinical scores is as follows: 1, ruffled fur/hunched back; 2, paresis/difficulty walking; 3, paralysis; 4, moribund/euthanized; and 5, dead. Error bars represent SEM. (C) The kinetics and levels of WNV were determined in the serum of the WT and *db/db* mice after WNV infection at indicated time-points by plaque assay. The data are expressed as PFU/mL of serum. Each data point represents an individual mouse, and data from two independent experiments are depicted. Data points below the horizontal dotted line are negative. *p<0.05, **p<0.001, ***p<0.0001.

To investigate the effect of diabetes on WNV pathogenesis, we evaluated the morbidity and mortality of WT and *db/db* mice after infection with PBS (mock) or 10 PFU of WNV. PBS inoculated mice remained healthy throughout the observation period of 21 days, whereas *db/db* mice were more susceptible to WNV disease and displayed significantly higher mortality rate as compared to WT mice, 92% vs. 37%, p<0.0001 (Fig. 2A). At day 21 after infection all surviving animals were confirmed to be positive for WNV IgG antibodies. As depicted in Fig. 2B, both *db/db* and WT mice demonstrated clinical evidence of infection characterized by ruffled fur and hunchbacked posture, however, neurological symptoms such as paresis, hind limb paralysis, tremors and ataxic gait were more severe in *db/db* mice and was observed in all the infected *db/db* mice as compared to approximately 50% of the WT mice.

The WNV replication kinetics in the serum of WT and *db/db* mice as measured by plaque assay demonstrated higher and more prolonged viremia in *db/db* mice. Virus titer was similar in WT and *db/db* mice at day 2 after infection (2×10^3^ PFU/mL), however at day 4 after infection, the virus titer in *db/db* mice was two logs higher than the WT mice, 2×10^5^ vs. 1.5×10^3^ PFU/mL, p<0.001 (Fig. 2C). At day 6 after infection, WNV levels decreased in WT mice, however it remained significantly high in *db/db* mice (1.1×10^3^ PFU/mL, p<0.001), suggesting sustained replication or delay in clearance of WNV from the periphery. The virus was cleared from the periphery of all *db/db* mice by day 8 after infection.

### Virus replication in the periphery and brain of *db/db* mice

**Figure 3:**
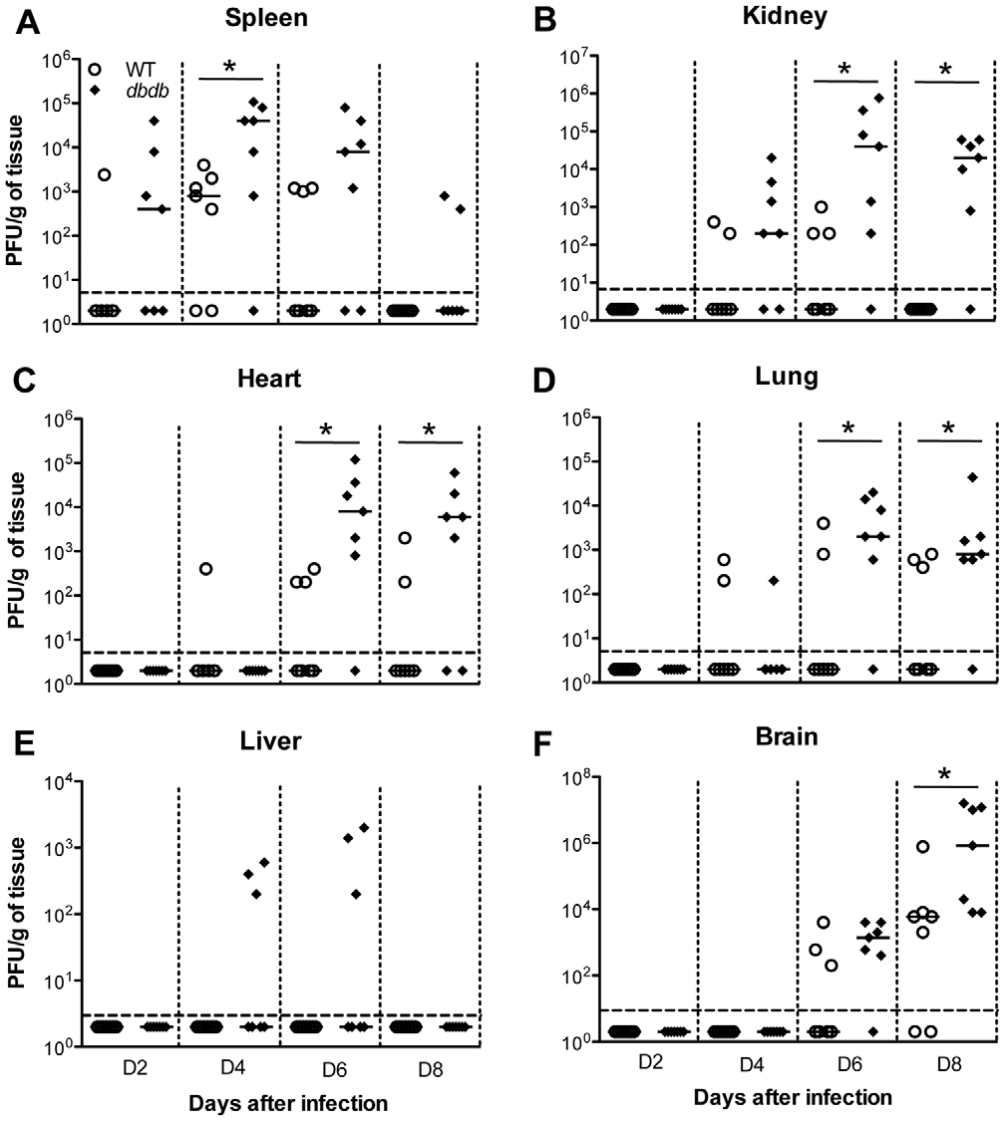
Viral burden and replication kinetics in the peripheral tissues and brain of WT and *db/db* mice. Nine-week old male WT and *db/db* mice were inoculated subcutaneously with 10 PFU of WNV, and the peripheral tissues and brains were harvested at days 2, 4, 6, and 8 after infection. Viral loads in the peripheral organs and brain were measured as noted in the figure by plaque assay using Vero cells and is reported as PFU per gram of tissue. Each data point represents an individual mouse, and data from two independent experiments are depicted. Data points below the horizontal dotted line are negative. The solid horizontal line signifies the median of seven mice per group. *p<0.05.

To understand how diabetes enhanced the susceptibility of mice to WNV disease, we compared WNV viral load in the peripheral tissues and brain of WT and *db/db* mice at days 2, 4, 6 and 8 after infection (Fig. 3A–E).

#### A) Spleen

Consistent with the earlier studies [22], [23], WNV was detected in the spleen of 1 of 7 WT mice at day 2 after infection (Fig. 3A). In contrast, 67% (4 of 7) of *db/db* mice had measurable virus titers (6×10^2^ PFU/g) at day 2 after infection. By day 4, which corresponds to the peak of WNV infection in spleen, significantly higher virus titer was observed in *db/db* mice when compared to the WT mice; 4×10^4^ vs. 8×10^2^ PFU/g, p<0.05. While WNV was cleared from the spleen of 4 of 7 WT mice at day 6 after infection, an elevated virus titer of 8×10^3^ PFU/g was detected in the *db/db* mice. The virus was cleared from all WT mice by day 8 after infection, however, in 2 of 7 *db/db* mice 8×10^2^ PFU/g of virus was detected.

#### B) Kidney

The kidney of WT mice is relatively resistant to WNV infection and high levels of virus is usually not detected [22], [23]. As expected, low levels of virus were detected in the kidneys of few WT mice at days 4 (2 of 7) and 6 (3 of 7) after infection (Fig. 3B). Nonetheless, significantly high WNV replication was detected in the kidneys of *db/db* mice at day 4 (2×10^2^ PFU/g) and day 6 (4×10^4^ PFU/g, p<0.05). However, unlike spleen, there was no clearance phase, as high levels of virus persisted in the kidneys of *db/db* mice at day 8 after infection; 2×10^4^ PFU/g, p<0.05.

#### C) Heart

WNV was undetectable in the hearts of both WT and *db/db* mice at days 2 and 4 after infection except one WT mice that was positive at day 4 (Fig. 3C). *db/db* mice developed heart infection beginning day 6 after infection with 85% of *db/db* mice exhibiting high viremia (8×10^3^ PFU/g, p<0.05), which persisted till day 8 (6×10^3^ PFU/g, p<0.05) after infection. In comparison, low levels of virus was detected in only 42% and 28% of WT mice at days 6 and 8 after infection, respectively.

#### D) Lung

Virus replication kinetics observed in the lung was similar to the heart. WNV burden in the lungs was either below the limit of detection or very low in all mice at days 2 and 4 after infection (Fig. 3D). While, WNV was detected only in 28% and 42% of WT mice at days 6 and 8 respectively, 85% of *db/db* mice had high viremia at day 6 (2×10^3^ PFU/g) and day 8 (8×10^2^ PFU/g) (p<0.05 for both time points) after infection.

#### E) Liver

Consistent with previous studies [31], no virus was detected in the liver of WT mice at any time point after infection (Fig. 3E). Unlike other peripheral organs, virus was also not detected in the liver of *db/db* mice at any time point with the exception of three *db/db* mice at days 4 and 6 after infection, suggesting an inability of virus to replicate efficiently in the liver.

#### F) Brain

Virus was not detected in the brain of both WT and *db/db* mice at days 2 and 4 after infection (Fig. 3F), which is consistent with the previous studies [22], [23]. At day 6 after infection, virus was detected in the brain of 85% (6 of 7) of *db/db* mice as compared to only 42% (3 of 7) of WT mice. *db/db* mice (7 of 7, 100%) continued to show significantly increased viral burden when compared to WT mice (5 of 7, 71%) on day 8 after infection, 8.4×10^4^ vs. 6×10^3^ PFU/g, p<0.05, with increased morbidity and mortality.

### Adipose tissues support the WNV replication in *db/db* mice

**Figure 4:**
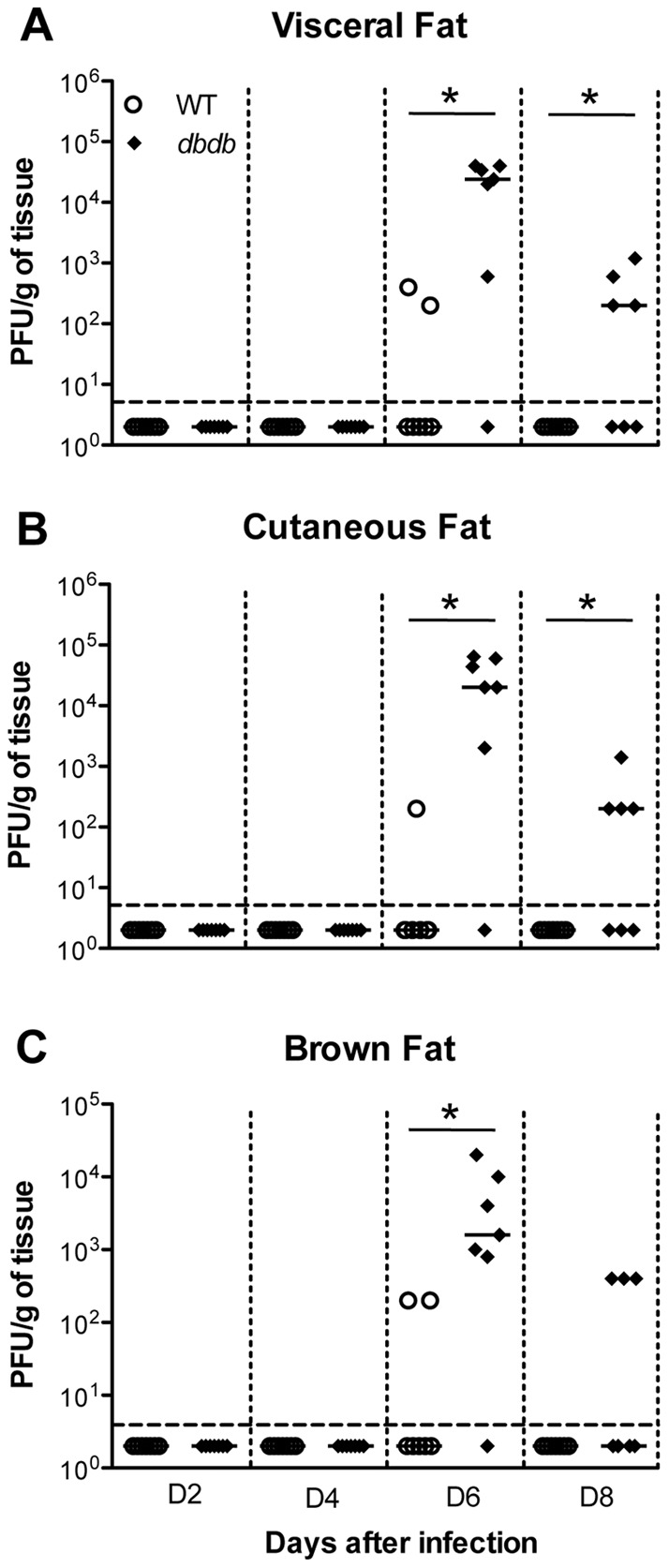
Viral load analysis in the adipose tissues of WT and *db/db* mice. Viral loads were determined in (A) visceral, (B) cutaneous and (C) brown fat in WT and *db/db* mice at days 2, 4, 6, and 8 after infection by plaque assay using Vero cells and is reported as PFU per gram of tissue. Each data point represents an individual mouse, and data from two independent experiments are depicted. Data points below the horizontal dotted line are negative. The solid horizontal line signifies the median of seven mice per group. *p<0.05.

Infectious virus was assayed in the white (visceral and cutaneous fat) and brown adipose tissues, to determine whether fat supports the WNV replication. Virus was undetectable in all the WT and *db/db* mice at days 2 and 4 after infection (Fig. 4). While, WNV was detected in only 25% of WT mice at day 6 after infection, 90% of *db/db* mice exhibited high virus titers in the adipose tissues (p<0.05). At day 8 after infection, 60% of *db/db* mice were positive for WNV, but virus was undetectable in all of the WT mice (p<0.05). In general, WNV levels were higher in white adipose tissue than brown adipose tissue.

### WNV-specific antibody responses in *db/db* mice

**Figure 5:**
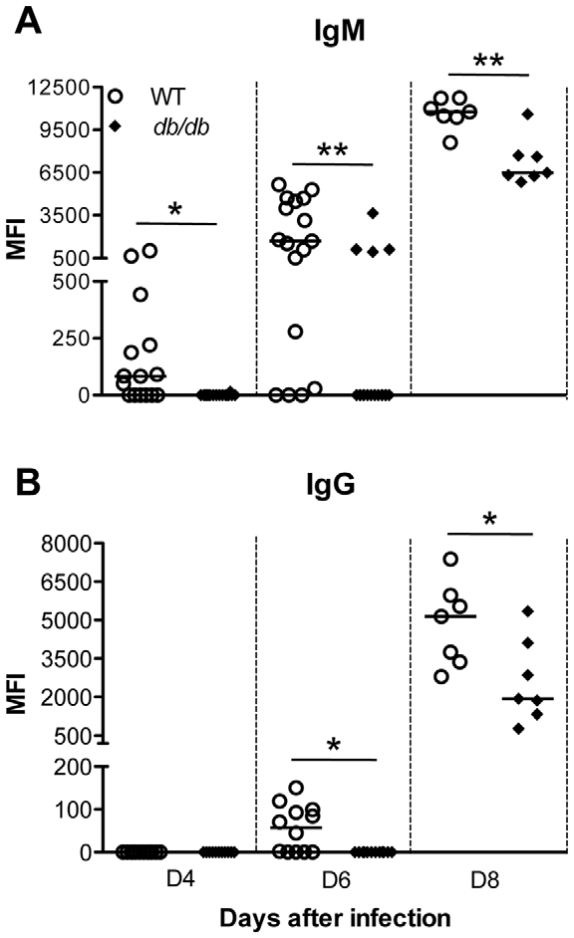
WNV-specific antibody responses in WT and *db/db* mice. Serum was collected from WT and *db/db* mice at indicated time points and WNV-specific (A) IgM and (B) IgG responses were measured by MIA using WNV E antigen as described in materials and methods. The values represent median fluorescent intensity (MFI) of individual infected mice minus mean MFI+3 standard deviation of respective mock group (n = 8). The data are depicted as scattered points representing individual mice with the median represented as the horizontal line and are representative of two independent experiments (n = 7–18). *p<0.05, **p<0.001.

Humoral immunity is an essential component of the immune response to WNV infection [23], [24]. Because we observed a high viremia and mortality in *db/db* mice we reasoned that this could be due to depressed WNV-specific antibody responses. Therefore, we next examined WNV-specific IgM and IgG antibodies profile in serum of WT and *db/db* mice using MIA. Consistent with previous studies [29], in WT mice WNV-specific IgM antibodies were first detected by day 4 after infection and gradually increased at days 6 and 8 after infection (Fig. 5A). In contrast, the development of WNV-specific IgM was delayed in *db/db* mice and exhibited significantly lower titers at days 4, 6 and 8 after infection (p<0.05 for day 4 and p<0.001 for days 6 and 8). Similar to IgM antibodies, overall levels of WNV-specific IgG antibodies were also significantly reduced in *db/db* mice as compared with WT mice at days 6 and 8 after infection (Fig. 5B, p<0.05).

### Antiviral IFN-α response in *db/db* mice after WNV infection

**Figure 6:**
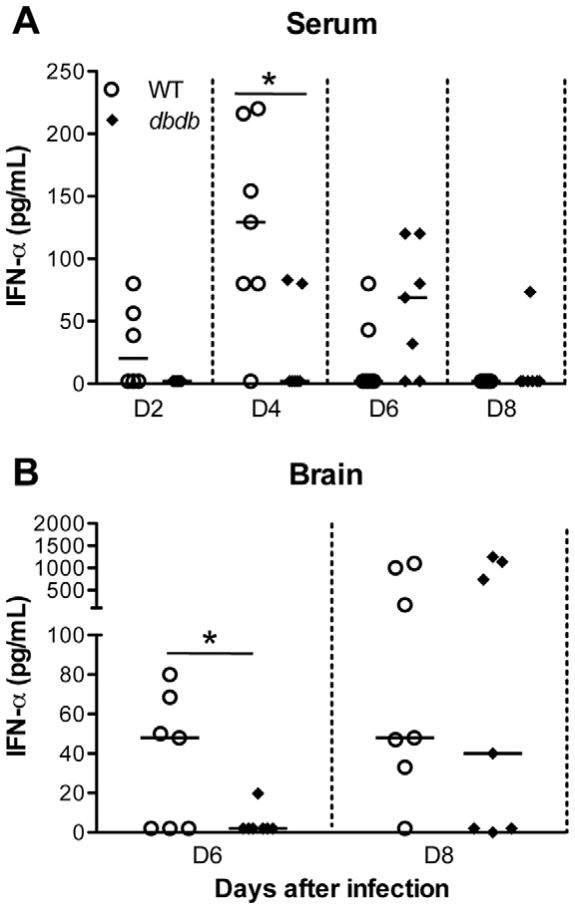
IFN-α levels in serum and brain of WT and *db/db* mice after WNV infection. (A) Serum was collected from WT and *db/db* mice at indicated days after infection. IFN-α production was measured using mouse IFN-α ELISA kit. (B) Brains were harvested from WNV-infected WT and *db/db* mice at days 6 and 8 after infection and homogenized as described in materials and methods. Data depicted are representative of two independent experiments. The solid horizontal line signifies the median of seven mice per group. *p<0.05.

Several studies provide evidence for the role of type 1 IFN in limiting WNV replication [22]. Because we observed high virus replication in periphery and brain and low levels of WNV-specific antibodies, we speculated that impaired interferon response in *db/db* mice might be the mechanism behind this phenomenon. Therefore, we measured the levels of IFN-α in the serum and brain of WT and *db/db* mice. IFN-α was first detected in WT mice at day 2 after infection, peaked at day 4 and then decreased at day 6 after infection (Fig. 6A). In contrast, *db/db* mice did not elicit IFN response at day 2 after infection and at day 4, the levels were significantly low in comparison to WT mice (p<0.05). High levels of IFN-α were not detected in *db/db* mice until day 6 after infection. We next examined IFN-α levels in the brain at days 6 and 8 after infection. In the brain WT mice developed a detectable interferon response at day 6 after infection, while there was no IFN-α detected in *db/db* mice at this time point (Fig. 6B, p<0.05). However, IFN-α levels were similar in the brains of WT and *db/db* mice at day 8 after infection.

### Inflammatory cytokine profile in *db/db* mice after WNV infection

**Figure 7:**
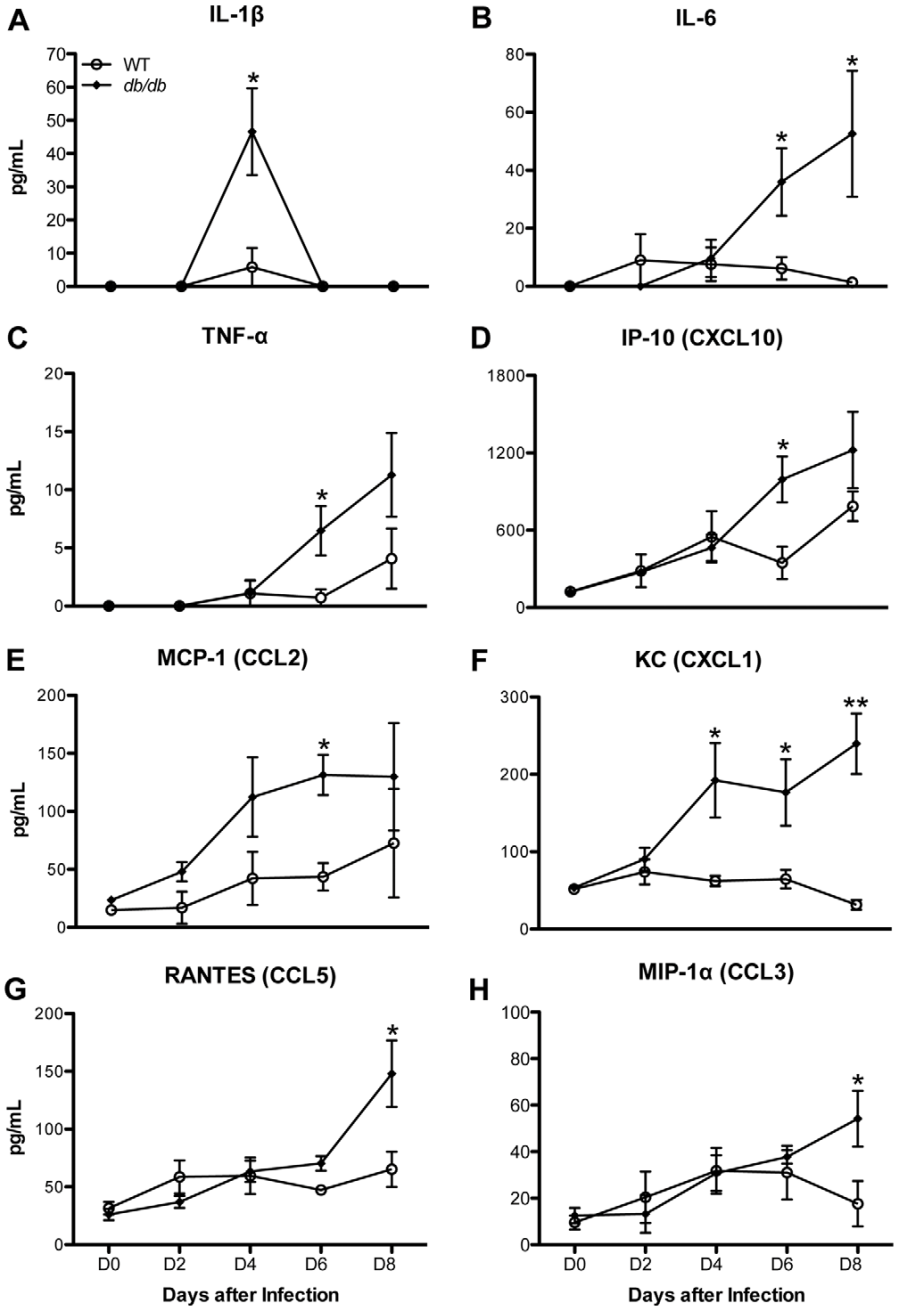
Cytokines and Chemokines levels in serum of WT and *db/db* mice after WNV infection. Serum was collected from WT and *db/db* mice at indicated days after infection. Levels of chemokines and cytokines as noted in the figure were measured using multiplex Luminex assay and are expressed as the mean concentration (pg/mL) ± SEM, representing two independent experiments (n = 7 per group). *p<0.05, **p<0.001.

Type 2 diabetes is associated with enhanced inflammatory response to infections [8], [9]. Therefore, we measured levels of multiple cytokines and chemokines in the serum of WT and *db/db* mice after WNV infection. As expected, the inflammatory response was more pronounced in the *db/db* mice than the WT mice. IL-1β levels increased sharply in *db/db* mice at day 4 after infection (Fig. 7A, p<0.05) and were undetectable at days 6 and 8. The IL-6 levels were significantly up-regulated in the *db/db* mice at days 6 and 8 after infection (Fig. 7B, p<0.05). Similarly, the TNF-α levels in the serum of *db/db* mice were significantly higher than that in the WT mice at day 6 after infection (Fig. 7C, p<0.05). A similar pattern was observed for chemokine expression. Both IP-10 and MCP-1 levels were significantly elevated in *db/db* mice at day 6 after infection (Fig. 7D and 7E, p<0.05). While, the levels of KC were significantly higher in *db/db* mice at days 4 through 6 after infection (Fig. 7F, p<0.05 for days 4 and 6 and p<0.001 for day 8), the levels of RANTES and MIP-1α (Fig. 7G and 7H, p<0.05) were only significantly increased at day 8 after infection when compared with the WT mice.

## Discussion

Type 2 diabetes is associated with an impaired immune response and increased susceptibility to various pathogens [3], [7]. However, studies examining the effects of diabetes on the immune response to viral infections are limited. To our knowledge, this is the first report characterizing the effect of diabetes on WNV infection and associated immune responses in a mouse model. In this study we demonstrate that WNV-infected *db/db* mice display high virus titers, increased tissue tropism, and high mortality rates in comparison to WT mice. These observations were associated with a significant delay in the induction of antiviral immune responses and increase in the pro-inflammatory responses in the *db/db* mice.

### Diabetes enhanced WNV replication, dissemination, and mortality

In *db/db* mice decreased survival rate was accompanied with increased and sustained WNV replication in the serum, peripheral tissues and brain. As compared to WT mice, *db/db* mice exhibited elevated levels of virus titers in the serum, which persisted until day 6 after infection and correlated with increased tissue tropism (Fig. 2C). While the spleen was the only peripheral organ with significant WNV infection in WT mice, *db/db* mice displayed significant virus replication in other peripheral tissues such as kidney, heart, and lung (Fig. 3). Similar to the periphery, there was enhanced virus replication in the brain of *db/db* mice leading to increased mortality. However, virus was not detected in the brain of surviving WT and *db/db* mice by plaque assay at day 21 after infection.

Several studies provide a link between diabetes and increased disease severity associated with multiple bacterial and parasitic pathogens. Infection of *db/db* mice with *Staphylococcus aureus* resulted in a prolonged infection with robust inflammatory response [5]. Similarly, infection with *Listeria monocytogenes* and *Trypanosoma cruzi* led to increased mortality and suppressed pathogen clearance in *db/db* mice [4], [6]. However, studies of virus infections in experimental diabetic models is limited except for coxsackievirus in which *db/db* mice displayed greater susceptibility to infection [32]. Our results suggest that in addition to multiple bacterial and parasitic pathogens, WNV infection also enhances disease severity among diabetics.

### WNV can efficiently replicate in adipose tissues of *db/db* mice

Little attention has been given to the role of adipose tissue in infectious diseases. Adipose tissue plays a major role in inflammation and is known to be a critical player in the pathogenesis of several infectious diseases [33]. The adipocyte can be a direct target for a number of pathogens and their products. Several viruses such as cytomegalovirus, adenoviruses-2 and -36, and Rous sarcoma virus, are able to infect adipocytes in vitro and induce an inflammatory response [34]. Subtypes of adenoviruses can persistently infect adipocytes and induce obesity [35]. Furthermore, adipose tissue also serves as a reservoir for recrudescent disease caused by infections with *Rickettesiae prowazekii* and *Trypanosoma cruzi* [36], [37]. Published data suggest that WNV, not only causes acute disease, but also can persist long term in humans [38] and animal models [39]. Persistence of WNV has been observed in various organs such as skin, kidney, brain and lymphoid tissues [39]. Herein, we demonstrate high levels of WNV in the adipose tissue of *db/db* mice (Fig. 4) suggesting that adipose tissues can also serve as a principal site for WNV replication and persistence in diabetics. Moreover, the production of inflammatory cytokines from WNV-infected adipose tissue may play a significant role in host defense mechanisms during WNV infection.

### WNV-specific immune responses are severely impaired and delayed in *db/db* mice

Robust induction of antiviral immune responses is critical for the control of WNV infection [40]. IFN-α is rapidly produced following WNV infection and is critical for controlling virus replication, and restricting tissue tropism [22]. We observed a robust increase in the levels of IFN-α in the serum and brain of WT mice, which correlated with virus clearance. In comparison, there was a significant delay in the induction of IFN-α response in the serum and brain of the *db/db* mice (Fig. 6). IFN-α levels were not detected in the serum of *db/db* mice until day 6 after infection. It is important to note that virus titers were higher in the serum of *db/db* mice until day 6 after infection, suggesting that type 1 IFN response is defective in *db/db* mice and might be responsible for delayed virus clearance in *db/db* mice. Similar to IFN-α response, previous studies have linked reduced WNV-specific antibody responses early during the course of infection with higher viremia, early spread to the CNS, and increased mortality [23], [24]. Similarly, we observed significantly delayed production of WNV-specific IgM and IgG antibodies in *db/db* mice when compared to WT mice (Fig. 5). This may be due to the delayed production of IFN-α, as type I IFN has been reported to enhance humoral immune responses by stimulating dendritic cells [41] as well as directly affecting B cells [42], [43]. Purtha et al. has also demonstrated that early B-cell activation after WNV infection requires α/β interferon [42]. Although, the impaired immune responses such as leukocyte activation, and cytokines and chemokines production have been characterized in diabetes models [3], [7], [8], the effect of diabetes on Type 1 IFN response have so far not been reported. This is the first report demonstrating the effect of diabetes on suppressing key antiviral defense responses such as those elicited by IFN-α, and IgM and IgG antibodies. However, Smith et al. have previously reported attenuated Type 1 IFN response in a related diet-induced obesity model upon infection with Influenza virus [44]. This finding also has significant implications on vaccine strategies for WNV in an increasingly diabetic population.

### Enhanced inflammatory response in *db/db* mice

Several studies have demonstrated that WNV-induced proinflammatory responses modulate BBB permeability, facilitate leukocyte penetration into the CNS, activate glial cells and mediate neuronal death after WNV infection [20], [21], [27]–[29]. In this study we demonstrate significantly elevated levels of potent cytokines and chemokines in the serum of *db/db* mice after WNV infection. The levels of IL-1β were high at day 4 after infection in *db/db* mice, which is also the peak of viremia. IL-1β is involved in WNV-induced Langerhans cell migration from the skin to draining lymph nodes in the mice model [45], and also plays a critical role in the pathogenesis of type 2 diabetes and its associated complications [9], [46]. IL-1β is a master regulator and modulates the secretion of other cytokines such as IL-6 and TNF-α [46]. It has been demonstrated that IL-1β-mediated inflammation is augmented in *db/db* mice due to diabetes-associated loss of IL-1β counter-regulation [47]. Similarly in our study, induction of IL-1β preceded the up-regulation of other inflammatory cytokines such as IL-6 and TNF-α, and chemokines such as IP-10 (CXCL10), KC (CXCL1), MCP-1 (CCL2), RANTES (CCL5), and MIP-1α (CCL3) in the serum of *db/db* mice at days 6 and 8 after infection (Fig. 7), which also correlated with the appearance of the WNV in various peripheral tissues and entry of the virus in the brain of *db/db* mice (Fig. 3). Similar to the serum profile of pro-inflammatory cytokines and chemokines, unpublished data from our laboratory demonstrate increased levels of these cytokines and chemokines in the brain of *db/db* mice at days 6 and 8 after infection (Kumar et. al., unpublished data), suggesting enhanced inflammatory response both in the periphery and brain of *db/db* mice after WNV infection. These data are consistent with previous observations that *db/db* mice demonstrate a greater inflammatory response to various pathogens such as *Staphylococcus aureus*, *Porphyromonas gingivalis*, and *Trypanosoma cruzi*, in which heightened inflammatory response was correlated with increased disease severity [5], [8], [48]. The increased inflammation observed in WNV-infected *db/db* mice could also have multiple effects, including enhanced virus and leukocyte entry into the brain, increased neuroinflammtion and neuronal death, thereby contributing to increased mortality.

In conclusion, *db/db* mice were highly susceptible to WNV disease and suppressed clearance of virus was observed in serum, peripheral tissues and brain. Innate and humoral immune responses are required for resistance to WNV infection and the impaired antiviral immune response in *db/db* mice might be involved in attenuated anti-WNV resistance. These findings provide direct experimental evidence of type 2 diabetes as a risk factor for severe WNVE. Further studies are warranted to elucidate the underlying mechanisms to develop adjunct therapeutics for diabetics presenting with WNVE symptoms.

## Materials and Methods

### Ethics statement

This study was specifically approved by the University of Hawaii Institutional Animal Care and Use Committee (IACUC) (protocol number 10-948), and conducted in strict accordance with guidelines established by the National Institutes of Health and the University of Hawaii IACUC. All animal experiments were conducted in consultation with veterinary and animal care staff at the University of Hawaii in animal biosafety level-3 laboratory, and mice that exhibited severe disease were euthanized to limit suffering.

### Animal experiments

Male nine-week old C57BL/6J-*Lepr^db^/Lepr^db^* (*db/db*) mice and C57BL/6J (WT) mice were purchased from The Jackson Laboratory and were acclimatized for 2–4 days in the animal biosafety level-3 laboratory prior to the start of the study. Animals were housed four per cage and allowed to eat and drink *ad libitum*. The animal suite was maintained at 72°F, 45% humidity and on 12/12 light/dark cycle. Sawdust bedding was provided along with paper towel and the cages were changed weekly. Trained and certified personnel conducted all the animal experiments.

For survival studies, mice were inoculated via the footpad route with 10 plaque forming units (PFU) of WNV (NY99) or PBS (mock), and the disease symptoms and mortality were observed for 21 days as described previously [49]. Clinical symptoms were observed twice a day as described previously [50]. These symptoms included ruffled fur, hunchbacked posture, difficulty walking, hind limb paralysis, tremors and ataxic gait. To limit suffering animals displaying severe symptoms such as tremors and ataxic gait, were euthanized immediately using CO_2_. On days 2, 4 and 6 after infection, 100 μL blood was collected from the tail vein, and serum was separated and frozen for analysis of WNV titer by plaque assay using Vero cells as described previously [51].

In a separate set of experiments WT and *db/db* mice were inoculated with PBS or 10 PFU of WNV and at days 2, 4, 6, and 8 after infection, mice were anesthetized using isoflurane and perfused with PBS. Spleen, kidneys, liver, heart, lungs, visceral fat, cutaneous fat, brown fat and brains were harvested and flash frozen in 2-methylbutane (Sigma) at aforementioned time points. Tissues were weighed and homogenized in a bullet blender (Next Advance) using glass or zicronium beads as per manufacturer's instructions, and plaque assay was conducted as described previously [50].

### Intraperitoneal glucose tolerance test (IGTT)

Mice were fasted for 4 h and IGTT was conducted as described previously [52]. Following a baseline (0 min) blood sample withdraw, 2 mg/g body weight of D-glucose was injected and the concentration of glucose was measured in duplicate samples at 30, 60, 90, and 120 min using One Touch Basic glucometers and One Touch glucose test strips [52].

### Measurement of WNV-specific antibodies

The levels of WNV-specific IgM and IgG antibodies were measured in the serum using microsphere immunoassay (MIA) for WNV envelope E protein as described previously [39]. Briefly, serum samples (1∶20 dilution) were incubated with the microspheres coupled with a recombinant WNV E antigen for 30 min followed by secondary goat anti-mouse IgG or IgM conjugated to red-phycoerythrin for 45 min. The fluorescence intensity of the microspheres was analyzed with a Luminex 100 instrument.

### ELISA

The levels of IFN-α were measured in the serum and brain homogenates by ELISA, using the *VeriKine^TM^* Mouse Interferon-Alpha ELISA Kit (PBL Interferon Source) as described previously [27].

### Measurement of Cytokines and Chemokines

The levels of cytokines and chemokines were measured in the serum by Luminex assay using MILLIPLEX MAP Mouse Cytokine/Chemokine kit (Millipore).

### Statistical analysis

Log-rank (Mantel-Cox) Test and Gehan-Breslow-Wilcoxon Test were used to analyze survival data. Mann-Whitney test and unpaired student t-test using GraphPad Prism 5.0 was used to calculate p values of difference between viral titers and immune responses, respectively. Differences of p<0.05 were considered significant.
